# Voltage-sensitive sodium channel (*Vssc*) mutations associated with pyrethroid insecticide resistance in *Aedes aegypti* (L.) from two districts of Jeddah, Kingdom of Saudi Arabia: baseline information for a *Wolbachia* release program

**DOI:** 10.1186/s13071-021-04867-3

**Published:** 2021-07-12

**Authors:** Nancy M. Endersby-Harshman, AboElgasim Ali, Basim Alhumrani, Mohammed Abdullah Alkuriji, Mohammed B. Al-Fageeh, Abdulaziz Al-Malik, Mohammed S. Alsuabeyl, Samia Elfekih, Ary A. Hoffmann

**Affiliations:** 1 PEARG, School of BioSciences, Bio21 Institute, The University of Melbourne, 30 Flemington Rd, Parkville, VIC Australia; 2 King Abdul-Aziz City for Science and Technology (KACST), Riyadh, Saudi Arabia; 3 CSIRO, Australian Centre for Disease Preparedness, Geelong, VIC Australia

**Keywords:** Dengue, Mosquito, Target-site, Knockdown resistance (*kdr*), Permethrin, Deltamethrin, DDT

## Abstract

**Background:**

Dengue suppression often relies on control of the mosquito vector, *Aedes aegypti*, through applications of insecticides of which the pyrethroid group has played a dominant role. Insecticide resistance is prevalent in *Ae*. *aegypti* around the world, and the resulting reduction of insecticide efficacy is likely to exacerbate the impact of dengue. Dengue has been a public health problem in Saudi Arabia, particularly in Jeddah, since its discovery there in the 1990s, and insecticide use for vector control is widespread throughout the city. An alternative approach to insecticide use, based on blocking dengue transmission in mosquitoes by the endosymbiont *Wolbachia*, is being trialed in Jeddah following the success of this approach in Australia and Malaysia. Knowledge of insecticide resistance status of mosquito populations in Jeddah is a prerequisite for establishing a *Wolbachia*-based dengue control program as releases of *Wolbachia* mosquitoes succeed when resistance status of the release population is similar to that of the wild population.

**Methods:**

WHO resistance bioassays of mosquitoes with deltamethrin, permethrin and DDT were used in conjunction with TaqMan^®^ SNP Genotyping Assays to characterize mutation profiles of *Ae*. *aegypti*.

**Results:**

Screening of the voltage-sensitive sodium channel (*V*ssc), the pyrethroid target site, revealed mutations at codons 989, 1016 and 1534 in *Ae*. *aegypti* from two districts of Jeddah. The triple mutant homozygote (1016G/1534C/989P) was confirmed from Al Safa and Al Rawabi. Bioassays with pyrethroids (Type I and II) and DDT showed that mosquitoes were resistant to each of these compounds based on WHO definitions. An association between *V*ssc mutations and resistance was established for the Type II pyrethroid, deltamethrin, with one genotype (989P/1016G/1534F) conferring a survival advantage over two others (989S/1016V/1534C and the triple heterozygote). An indication of synergism of Type I pyrethroid activity with piperonyl butoxide suggests that detoxification by cytochrome P450s accounts for some of the pyrethroid resistance response in *Ae*. *aegypti* populations from Jeddah.

**Conclusions:**

The results provide a baseline for monitoring and management of resistance as well as knowledge of *V*ssc genotype frequencies required in *Wolbachia* release populations to ensure homogeneity with the target field population. *V*ssc mutation haplotypes observed show some similarity with those from *Ae*. *aegypti* in southeast Asia and the Indo-Pacific, but the presence of the triple mutant haplotype in three genotypes indicates that the species in this region may have a unique population history.

**Graphical Abstract:**

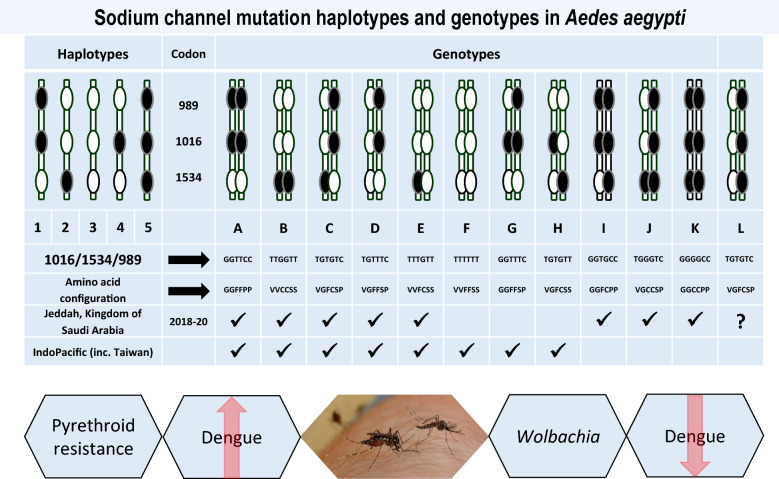

**Supplementary Information:**

The online version contains supplementary material available at 10.1186/s13071-021-04867-3.

## Background

Target-site resistance to pyrethroids in *Aedes aegypti*, also known as knockdown resistance (*kdr*), is an autosomal, incompletely recessive trait [1]. Voltage-sensitive sodium channel (*Vssc*) mutations at codons 1016 and 1534 occur in *Ae*. *aegypti* within the pyrethroid receptor sites in Domains II (S6) and III (S6) of the protein molecule [2]. A third mutation, S989P, which is often in perfect linkage with V1016G, is not known to reduce the sensitivity of the sodium channel [2], but confers some additive pyrethroid resistance in the homozygous state in combination with 1016G [3].

Pyrethroid resistance and *Vssc* mutations have been identified in *Ae*. *aegypti* from the Kingdom of Saudi Arabia and discussed in several studies [4–7]. Three mutation sites are being screened routinely in samples of *Ae*. *aegypti* from Jeddah and are labeled as S989P, V1016G and F1534C according to the codon numbering in the sequence of the most abundant splice variant of the house fly, *Musca domestica*, *Vssc* (GenBank accession nos. AAB47604 and AAB47605) [8]. However, direct links between these mutations and resistance phenotypes in this locality are still unclear.

Dengue has been a public health problem in Saudi Arabia and particularly in Jeddah since its discovery there in 1994 [9]. Dengue incidence is confined to the western and south western regions of Saudi Arabia, and cases have increased in Jeddah and Makkah from 2008 to 2012, possibly as a result of increased stagnant water due to construction, but also because of international travel associated with the Hajj [10]. Dengue cases peaked in 2013 with 6512 cases, and central Jeddah has been identified as a site of major dengue concentration [10]. Currently, dengue suppression relies on control of the mosquito vector, *Ae*. *aegypti*, through the applications of insecticides of which the pyrethroid group has played a dominant role [5]. Bifenthrin (Type I) and alpha-cypermethrin (Type II) are pyrethroids that have commonly been used in Jeddah.

Insecticide resistance is prevalent in *Ae*. *aegypti* around the world [11, 12], and the resulting reduction of insecticide efficacy is likely to be exacerbating the impact of dengue and of chikungunya, a second flavivirus that circulates in Saudi Arabia [10]. Insecticide resistance threatens the long-term utility of vector control in this region. Hence, an alternative approach based on blocking dengue transmission in mosquitoes by the endosymbiont *Wolbachia* is being trialed following the success of this approach in suppressing dengue in other countries [13, 14]. Preparation for a release of *Wolbachia*-infected mosquitoes for control of dengue transmission has commenced in the Al Safa and Al Rawabi districts of Jeddah in the Kingdom of Saudi Arabia. Aljamaa Municipality, in which Al Rawabi is located, had the highest numbers of both *Ae*. *aegypti* and *Culex* mosquitoes out of all Municipalities of Jeddah over a 2-month collection period in 2015–2016 [15]. Al Safa is located in the Almatar Municipality, where moderate to high numbers of *Ae*. *aegypti* were caught in the same study [15].

Knowledge of the insecticide resistance status of mosquito populations in Jeddah is one prerequisite for establishing a *Wolbachia*-based dengue control program. The presence and frequency of the mutants can be used as a measure of resistance in the local population of *Ae*. *aegypti* that can be compared with *Wolbachia*-infected mosquitoes destined for release. Mosquitoes are screened for mutations in the *Vssc*, the target site of pyrethroid insecticides and DDT [16], to characterize the population from the field for comparison with the future population for release. Similar releases of *Wolbachia* mosquitoes have been shown to succeed when insecticide resistance status of the release population is similar to that in the field [13, 17] and to fail or strike difficulties if the release population of mosquitoes does not carry equivalent resistance traits [18, 19]. Although a *Wolbachia* program is not based on insecticide use, it is a reality that released mosquitoes will be exposed to insecticides in the field and therefore must initially be able to survive in such an environment.

In this study, we aimed:

1. To characterize the *Vssc* mutations 989/1016/1534 and haplotypes in the *Ae*. *aegypti* populations in Jeddah in two areas (Al Safa and Al Rawabi) to compare with known haplotype patterns in other parts of the world.
2. To compare the distribution of these *Vssc* mutations in dead and surviving mosquitoes from bioassays with insecticides that target the *Vssc* (Type I and Type II pyrethroids as well as DDT, which shows a similar mode of action to Type I pyrethroids [20]).
3. To screen for the presence of a fourth mutation at codon 410 known to confer pyrethroid resistance to *Ae*. *aegypti* in a restricted number of locations [21–23] and, if found, to characterize this mutation in bioassay dead and surviving mosquitoes by sequencing a region of *Vssc* Domain I where this mutation may be located.
4. To screen for a mutation in codon 1763, known from *Ae*. *aegypti* in Taiwan [24, 25], which does not affect the sensitivity of the *Vssc* [2], but may play a similar role to S989P in enhancing resistance conferred by the other mutations [24].
5. To use a synergist bioassay to look for evidence of a metabolic component of pyrethroid resistance in *Ae*. *aegypti* from Jeddah.

Knowledge about insecticide resistance in *Ae*. *aegypti* from the potential *Wolbachia* release sites is needed to ensure that similar traits are found in the strains of mosquitoes to be released to facilitate their survival under continued pressure of insecticide applications. Comparison of *Vssc* resistance haplotypes with those found in *Ae*. *aegypti* in other parts of the world gives preliminary information about population history of the species in Saudi Arabia. This knowledge will benefit the planned *Wolbachia* release programs and the development of long-term insecticide resistance management strategies for Jeddah.

## Methods

### Sample collection

**Fig. 1:**
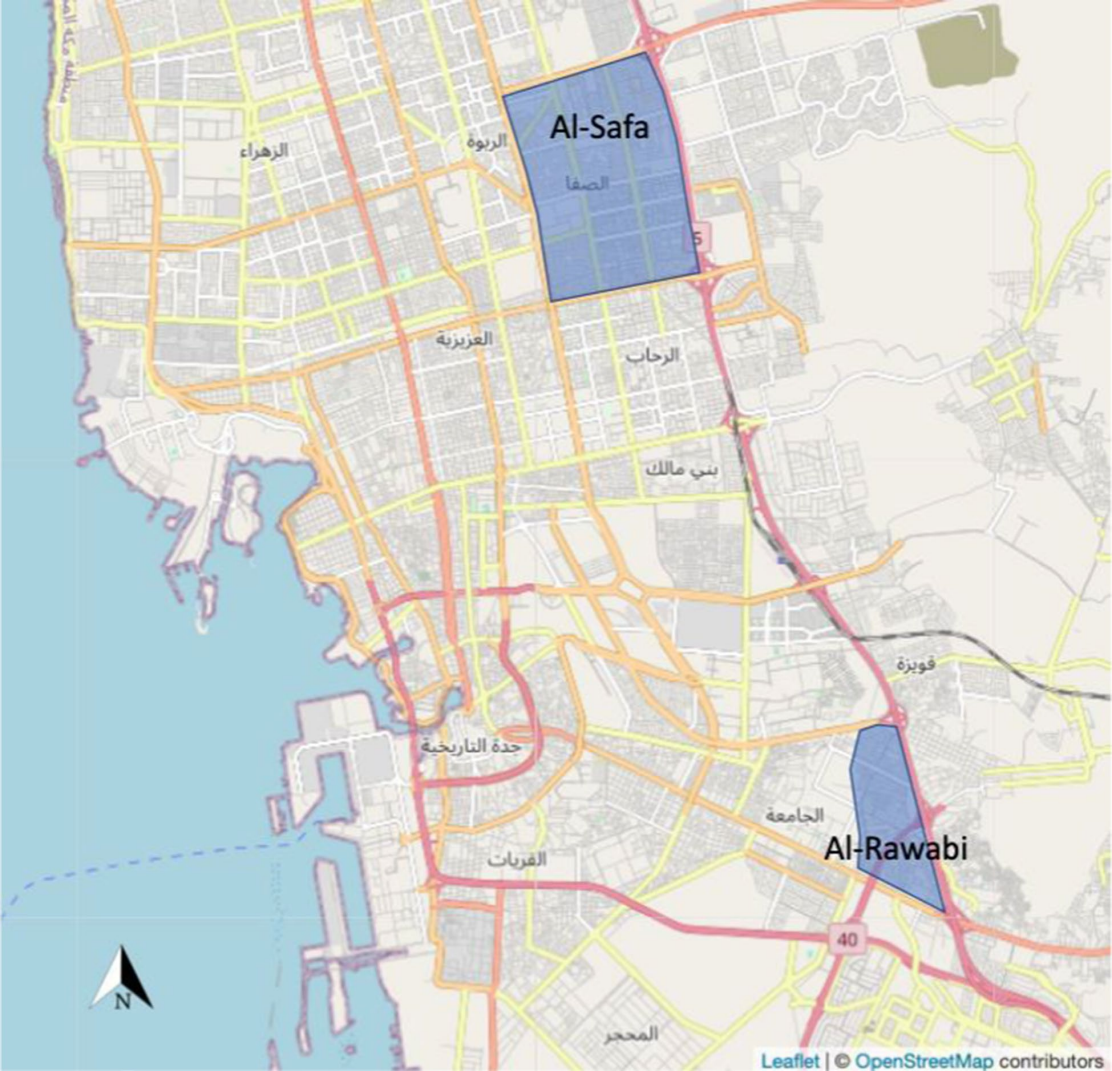
Map of Jeddah, Kingdom of Saudi Arabia, showing two mosquito sampling districts, Al Safa-9 and Al Rawabi

Two study districts were chosen for their status as dengue hotspots with large populations of *Ae*. *aegypti*, along with physical attributes such as highway boundaries to facilitate implementation of a *Wolbachia* program. Mosquito eggs were sampled in Al Safa-9 district (21.58343 N, 39.20830 E) located in central Jeddah and Al Rawabi district (21.47574 N, 39.26798 E) in the southern part of the city (Fig. 1) on three occasions (August 2018, October 2019 and February 2020) using oviposition bucket traps. The field sampling was performed over a 2-week period for each operation, and felt pieces containing eggs were collected twice and brought to the laboratory for rearing in separate containers, from which we tested an average of 10–12 mosquito samples (larval and adult stages, F_0_). These samples were used for screening the field populations for *Vssc* mutations.

Mosquito colonies were initiated and maintained in the Jeddah laboratory using larvae collected from various collection points in the Safa-9 district. Mosquitoes (F_0_) from this wild-type colony were used for WHO bioassays. Mosquito rearing was conducted at room temperature (26–27 °C) with around 70% relative humidity. Larvae were fed with fish food (Hikari Sinking Wafers^®^ Item # 21503, Kyorin Food Industries, Ltd., Japan).

### WHO bioassays

The standard WHO insecticide resistance bioassay involving insecticide-impregnated papers was used for adult mosquitoes [26, 27]. Twenty-five female adults aged < 5 days post-eclosion and non-blood fed were used as the test subjects. Five replicates and one control were used for each bioassay. Controls comprised the relevant solvent used for the insecticide papers (silicone oil for pyrethroids). The mosquitoes were exposed in WHO tubes to impregnated paper with diagnostic concentrations of deltamethrin (0.05%), permethrin (0.75%) or DDT (4%). Bioassays to test for synergism of permethrin 0.75% were conducted by exposing mosquitoes to piperonyl butoxide (PBO) (4%) for 1 h followed by exposure to permethrin (0.75%) for an additional hour. Insecticide impregnated papers were purchased from the Vector Control Research Unit, Universiti Sains Malaysia, Penang. Knockdown was scored every 10 min for a 1-h exposure period at room temperature (26–27 °C). After exposure, the mosquitoes were transferred into clean tubes and were supplied with cotton soaked in 10% sugar solution. Mortality was recorded after 24 h. Classification of resistance status was made based on pre-determined WHO guidelines in which mortality of < 90% characterizes a resistant population. Bioassays were repeated until at least 40 dead and 40 survivors could be collected for each insecticide to be used for analysis of *Vssc* mutations. Mosquitoes from the bioassays were stored in absolute ethanol at − 20 °C prior to *Vssc* screening.

### DNA extraction

DNA was extracted from adult mosquitoes or late instar larvae using the DNeasy^®^ Blood and Tissue kit (QIAGEN Sciences, Germantown, MD, USA), the Roche High Pure PCR template kit (Roche Molecular Systems, Inc., Pleasanton, CA, USA) according to the instructions of the manufacturer or Chelex^®^ 100 resin (Bio-Rad Laboratories Inc., Hercules, CA, USA). Two final elutions of DNA from field mosquitoes (reared from field-collected eggs, F_0_) were made from the kit extractions with the first being used for construction of genomic libraries for a related study and the second being used for screening of *Vssc* mutations after being diluted 1:5 with water. The same dilution factor was used for DNA extracted using Chelex^®^ 100 resin on samples which were not to be used for construction of genomic libraries (dead and surviving adult mosquitoes from insecticide bioassays, reared from field collected larvae, F_0_).

### Screening of *Vssc* mutations

#### 989/1016/1534

Custom TaqMan^®^ SNP Genotyping Assays (Life Technologies, Carlsbad, CA, USA) were developed for each of the three target site mutations (codons 989, 1016, 1534) and were run on the Roche LightCycler^®^ 480 [28]. Endpoint genotyping was conducted using the Roche LightCycler^®^ 480 Software, version 1.5.1.62.

One hundred thirty mosquito samples were screened from each of the two districts of Jeddah; 117 dead and 121 surviving mosquitoes were screened from insecticide bioassays (Table 2).

#### 410 and 1763

A subset of 32 samples was screened for a mutation at codon 410 in Domain I of the *Vssc* and 93 samples for codon 1763 in Domain IV by PCR and Sanger sequencing. Primers used to amplify the region around codon 410 (exon 10 in Domain I) of the *Vssc* were aegSCF10 (5ʹ-GTGTTACGATCAGCTGGACC-3ʹ and aegSCR10 (5ʹ-AAGCGCTTCTTCCTCGGC-3ʹ) from Tancredi et al. [29]. Primers to amplify codon 1763 in *Vssc* Domain IV were albSCF6 (5ʹ-TCGAGAAGTACTTCGTGTCG-3ʹ) and albSCR8 (5ʹ-AACAGCAGGATCATGCTCTG-3ʹ) [30]. Amplicons were sequenced with albSCF7 (5ʹ-AGGTATCCGAACGTTGCTGT-3ʹ) by Macrogen Inc., South Korea for Sanger sequencing.

A 25-µl PCR master mix was set up as follows: 2.5 µl Standard Taq (Mg-free) Reaction Buffer (10×) (B9015 New England BioLabs Inc., Ipswich, MA, USA), 2.0 µl dNTP mix (10 mM total) (Bioline (Aust) Pty, Ltd, Eveleigh, NSW, Australia), MgCl_2_ (50 mM) (Bioline (Aust) Pty Ltd, Eveleigh, NSW, Australia), 1.25 µl of each primer (10 µM) (aegSCF10 and aegSCR10), 0.125 µl IMMOLASE™ DNA Polymerase (5 u/µl) (Bioline (Aust) Pty Ltd, Eveleigh, NSW, Australia), 15.125 µl PCR-grade water and 2 µl DNA (1:10 dilution of Chelex^®^-extracted DNA). A PCR reaction of 95 °C for 10 min, 35 cycles of 95 °C 30 s, 55 °C 45 s, 72 °C 45 s and a final extension at 72 °C for 5 min was run on an Eppendorf Mastercycler^®^ (Eppendorf AG, Hamburg, Germany). Amplicons were observed on a 1.5% agarose gel (Bioline (Aust) Pty, Ltd, Eveleigh, NSW, Australia) run for 40 min at 90 V stained with SYBR™ Safe DNA Gel Stain (Thermo Fisher Scientific Inc., Waltham, MA, USA) and viewed with a GelDoc XR Gel Documentation System (Bio-Rad Laboratories Inc., Hercules, CA, USA). Samples were sent to Macrogen Inc., South Korea, for Sanger sequencing. Amplicons for the codon 410 screen were sequenced with aegSCR10, and those for codon 1763 were sequenced with albSCF7 (5ʹ-AGGTATCCGAACGTTGCTGT-3ʹ). Sequences were analyzed with Geneious Prime Version 2020 (Biomatters Ltd.).

Samples screened were those collected in Al Rawabi and Al Safa in February 2020. Sequences were first aligned to KY747529 [21] for verification as *Aedes aegypti Vssc* and then to KY747530.1 for codon 410 (mutant L) [21] and MK495874 (wildtype D) and MK495875 (mutant Y) [25] for codon 1763 for identification of a possible mutation.

### Statistical analyses

*Vssc* mutation data from *Ae*. *aegypti* from Al Safa and Al Rawabi collected over 3 years were analyzed for site differences in genotype frequencies using contingency tables with significance tested through the Chi-squared statistic. Monte Carlo tests were used to determine significance because expected values in some cells were particularly low, even when data were combined across collection dates. These analyses were performed in IBM SPSS Statistics (IBM Corp., Armonk, NY, USA; 2013).

Odds ratios (with 95% confidence intervals) [31] were calculated to indicate the odds of an individual surviving exposure to an insecticide if it carried one particular genotype compared with another. An odds ratio of 1 indicates that there is no relationship between survival and the genotype under investigation. If 95% confidence intervals of the odds ratio do not span the value “1” then this suggests that the genotype is associated with survival.

## Results

### *Vssc* mutations in temporal field samples

**Fig. 2:**
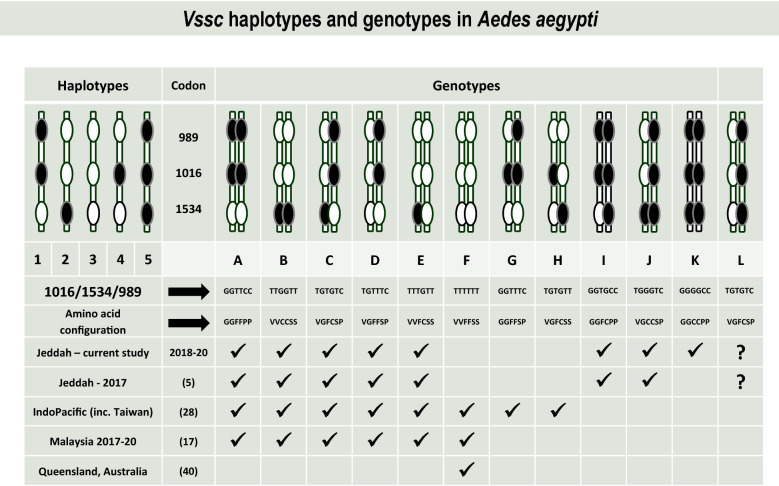
* Vssc* haplotypes and genotypes in *Aedes aegypti* from Jeddah from the current study and [5] compared with those found in southeast Asia [17] and the Indo-Pacific [28], including Australia [39]

**Fig. 3:**
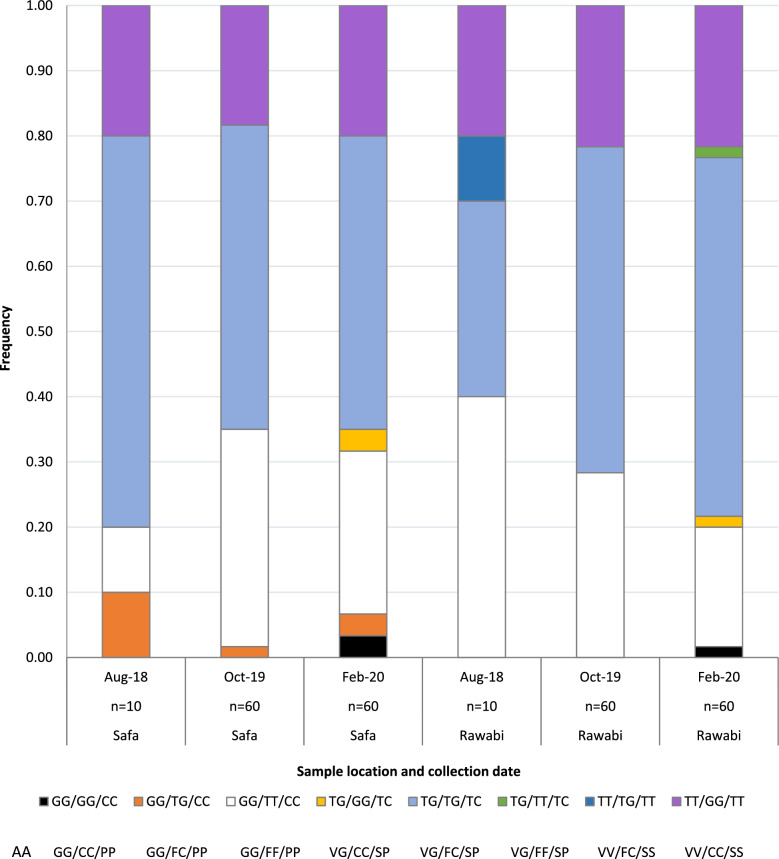
Comparison of frequency of *Vssc* genotypes in *Aedes aegypti* from Al Safa and Al Rawabi districts, Jeddah, Kingdom of Saudi Arabia, 2018–2020 (order of mutations 1016/1534/989, T = wildtype, G or C = mutant; AA = amino acid configuration)

Data from multiple studies of *Ae*. *aegypti* [28, 32–35] suggest that certain haplotypes of the three mutation sites predominate in a population, and there is little evidence of crossing over to disrupt the phase patterns found. Four haplotypes and eight genotypes were found overall in the samples of *Ae*. *aegypti* tested from Jeddah (Fig. 2). An initial sample of ten individuals per district identified four *Vssc* genotypes (A, B, C, E) seen in samples from Asia and the Indo-Pacific (Table 1, Fig. 3). One individual from Safa had a unique genotype (a homozygous mutant at codon 1016 and 989, but a heterozygote at 1534—genotype I) (Table 1). The linkage patterns we have observed for these mutations in mosquitoes from Asia and the Indo-Pacific cannot produce individuals of genotype I found in Safa (Fig. 2). To obtain this genotype, one parent had to contribute a triple mutant haplotype (H5), and the other contributed a second haplotype (H1) that we see in Asia/Indo-Pacific samples (Fig. 2).

**Table 1:** Voltage-sensitive sodium channel (*Vssc*) mutations in *Aedes aegypti* from Al Safa and Al Rawabi districts, Jeddah, 2018, 2019, 2020 (order of mutations is 1016/1534/989, upper genotype is the base configuration: T = wildtype, G or C = mutant; lower genotype is the amino acid configuration)

Genotype		Safa		Safa		Safa		Rawabi		Rawabi		Rawabi	
		Aug-18		Oct-19		Feb-20		Aug-18		Oct-19		Feb-20	
		*n*	Freq	*n*	Freq	*n*	Freq	*n*	Freq	*n*	Freq	*n*	Freq
GG/GG/CC GG/CC/PP	K	0	0.00	0	0.00	2	0.03	0	0.00	0	0.00	1	0.02
GG/TG/CC GG/FC/PP	I	1	0.10	1	0.02	2	0.03	0	0.00	0	0.00	0	0.00
GG/TT/CC GG/FF/PP	A	1	0.10	20	0.33	15	0.25	4	0.40	17	0.28	11	0.18
TG/GG/TC VG/CC/SP	J	0	0.00	0	0.00	2	0.03	0	0.00	0	0.00	1	0.02
TG/TG/TC VG/FC/SP	C/L	6	0.60	28	0.47	27	0.45	3	0.30	30	0.50	33	0.55
TG/TT/TC VG/FF/SP	D	0	0.00	0	0.00	0	0.00	0	0.00	0	0.00	1	0.02
TT/TG/TT VV/FC/SS	E	0	0.00	0	0.00	0	0.00	1	0.10	0	0.00	0	0.00
TT/GG/TT VV/CC/SS	B	2	0.20	11	0.18	12	0.20	2	0.20	13	0.22	13	0.22
Total		10		60		60		10		60		60	

Test for site differences (all dates combined) *χ*^2^ = 7.27, *df* = 7, *P* = 0.401 (0.388–0.413, 99% confidence intervals)

The second screen consisted of 60 samples from each study site (Al Rawabi and Al Safa Districts, Jeddah), collected in October 2019. Three of the *Vssc* genotypes (A–C—Fig. 2) found in the mosquitoes collected from these sites in Saudi Arabia were those found in samples from the Indo-Pacific region [28]. However, there was again one individual from Al Safa with genotype I (Table 1).

The third sample of *Ae*. *aegypti* was taken in February 2020, and again 60 samples from each site were screened for *Vssc* mutations. Genotype I was again found in mosquitoes from Al Safa (Table 1). Genotype J, which also contains the triple mutant haplotype (Fig. 2), was found in both Al Safa and Al Rawabi, and triple mutant homozygotes (genotype K—Fig. 2) were found in both Al Safa (two individuals) and Al Rawabi (one individual) (Fig. 3). Two genotypes found only in the Al Rawabi sample (D and E—Fig. 2—one individual of each) indicate that the triple wildtype haplotype is present in that district, but was not found as a homozygote. The frequency of each of the common genotypes (A, B, C) is similar between Al Safa and Al Rawabi (Table 1), but there are differences in distribution of the rare genotypes (Fig. 3). When data for all sampling dates were combined, there was no significant difference in genotypes between mosquitoes sampled in Al Safa and Al Rawabi (*χ*^2^ = 7.27, *df* = 7, *P* = 0.401, 0.388–0.413, 99% confidence intervals).

### WHO bioassays

1. Mortality
  None of the bioassays conducted showed mortality in the range 98–100%, which would indicate susceptibility according to WHO guidelines [36]. Permethrin 0.75% induced only 44.8% mortality, and DDT 4% bioassays showed mortality ranging from 3.2 to 10.4%, confirming a high level of resistance to these compounds in *Ae*. *aegypti* from Jeddah. Four bioassays with deltamethrin 0.05% showed an average mortality just under 90%, which indicates that the population of *Ae*. *aegypti* from Jeddah is resistant to this compound as well (Table 2).
2. Synergist bioassay
  Odds of mosquitoes being alive if exposed to permethrin 0.75% alone are just over twice those if exposed to permethrin 0.75% + PBO 4% (Table 3). PBO 4% alone caused some mortality, but the odds of being alive are not greater if exposed to permethrin compared with exposure to PBO alone (Table 3).
3. Comparison of *Vssc* genotypes in dead and survivors
  Five *Vssc* genotypes (A, B, C/L, J, K) were identified in the surviving pool of mosquitoes screened, and four genotypes (A, B, C/L, J) occurred in the dead mosquitoes exposed to 0.75% permethrin (Table 4). Two triple mutants were found in the survivors. There was no significant difference (α = 0.05) in the odds of being alive after exposure to permethrin 0.75% if carrying one genotype over any other (Additional file 1: Table S1).

**Table 2:** WHO bioassays of *Aedes aegypti* from Jeddah, KSA using permethrin 0.75%, deltamethrin 0.05%, DDT 4%—% mortality and numbers in *Vssc* screen

Date	Status	Insecticide	Tube 1	Tube 2	Tube 3	Tube 4	Tube 5	Total	Control	% mortality	*Vssc* screen
26-Sep-20	Dead	Permethrin 0.75%	10	8	17	13	8	56	0	44.8	40
26-Sep-20	Alive	Permethrin 0.75%	15	17	8	12	17	69	25		40
2-Sep-20	Dead	Deltamethrin 0.05%	16	17	18	18	15	84	0	84.0	10
2-Sep-20	Alive	Deltamethrin 0.05%	4	3	2	2	5	16	20		16
8-Sep-20	Dead	Deltamethrin 0.05%	20	18	18	18	19	93	1	92.5^a^	10
8-Sep-20	Alive	Deltamethrin 0.05%	0	2	2	2	1	7	19		7
15-Sep-20	Dead	Deltamethrin 0.05%	17	19	19	18	19	92	0	92.0	10
15-Sep-20	Alive	Deltamethrin 0.05%	3	1	1	2	1	8	25		8
17-Sep-20	Dead	Deltamethrin 0.05%	19	18	17	18	18	90	0	90.0	10
17-Sep-20	Alive	Deltamethrin 0.05%	1	2	3	2	2	10	20		10
26-Sep-20	Dead	DDT 4%	2	2	3	2	4	13	0	10.4	8
26-Sep-20	Alive	DDT 4%	23	23	22	23	21	112	25		10
7-Nov-20	Dead	DDT 4%	2	3	2	3	2	12	0	9.6	12
7-Nov-20	Alive	DDT 4%	23	22	23	22	23	113	25		10
8-Nov-20	Dead	DDT 4%	1	1	0	1	2	5	0	4.0	7
8-Nov-20	Alive	DDT 4%	24	24	25	24	23	120	25		10
9-Nov-20	Dead	DDT 4%	0	1	2	0	1	4	0	3.2	4
9-Nov-20	Alive	DDT 4%	25	24	23	24	24	120	25		10
12-Nov-20	Dead	DDT 4%	1	2	1	0	1	5	0	4.0	6
12-Nov-20	Alive	DDT 4%	24	23	24	25	24	120	25		0
14-Nov-20	Dead	DDT 4%	2	3	2	1	2	10	0	8.0	0
14-Nov-20	Alive	DDT 4%	23	22	23	24	23	115	25		0

^a^Corrected for control mortality using Abbott’s [40] formula

**Table 3:** WHO bioassays with permethrin 0.75% and synergist, piperonyl butoxide (PBO) and *Aedes aegypti* from Jeddah, KSA (*OR* odds ratio, *α* = 0.05, * = significance)

Date	Status	Permethrin 0.75%	PBO 4% only	Insecticide + PBO 4%	Solvent control
5-Nov-20	Dead	8	3	13	0
5-Nov-20	Alive	17	22	12	75
5-Nov-20	Dead	9	5	14	0
5-Nov-20	Alive	16	20	11	75
26-Oct-20	Dead	20	2	23	0
26-Oct-20	Alive	5	23	2	75
Total	Dead	37	10	50	0
	Alive	38	65	25	225
	% mortality	49.3	13.3	66.7	0.0

	*OR*	Lower c.i	Upper c.i	*α* = 0.05
Permethrin + PBO/permethrin	0.49	0.25	0.94	
Permethrin + PBO/PBO	0.08	0.03	0.17	
Permethrin/PBO	0.16	0.07	0.35	
Permethrin/permethrin + PBO	2.05	1.06	3.97	*
PBO/permethrin + PBO	13.00	5.72	29.54	*
PBO/permethrin	6.33	2.83	14.16	*

**Table 4:** Frequency (%) of *Vssc* mutations in dead and surviving *Ae*. *aegypti* mosquitoes from Jeddah from WHO bioassays with permethrin (0.75%), deltamethrin (0.05%) or DDT (4%) (order of mutations is 1016/1534/989, upper genotype is the base configuration: T = wildtype, G or C = mutant; lower genotype is the amino acid configuration; letters in parentheses refer to genotype code assigned in Fig. 2)

Insecticide	*Vssc* genotype					
	GG/TT/CC GG/FF/PP	TT/GG/TT VV/CC/SS	TG/TG/TC VG/FC/SP	GG/TG/CC GG/FC/PP	TG/GG/TC VG/CC/SP	GG/GG/CC GG/CC/PP
	(A)	(B)	(C/L)	(I)	(J)	(K)
Permethrin 0.75%						
Alive (*n* = 40)	30.0	12.5	50.0	0.0	2.5	5.0
Dead (*n* = 40)	27.5	30.0	40.0	0.0	2.5	0.0
Deltamethrin 0.05%						
Alive (*n* = 41)	43.9	19.5	31.7	4.9	0.0	0.0
Dead (*n* = 40)	12.5	27.5	57.5	0.0	2.5	0.0
DDT 4%						
Alive (n = 40)	25.0	32.5	32.5	7.5	2.5	0.0
Dead (n = 39)	17.9	28.2	41.0	2.6	10.3	0.0

Survivors of deltamethrin 0.05% fell into four genotypes as did the dead individuals, though the fourth and least frequent genotype differed between them (I for alive and J for dead) (Table 4). Odds ratios were significant (*α* = 0.05) for two genotype comparisons indicating that the odds of surviving were higher for individuals of genotype A than genotype B and also higher for genotype A than the triple heterozygote (genotype C or L) (Table 5).

**Table 5:** Odds of *Ae*. *aegypti* surviving with one *Vssc* genotype compared with another 24 h after a 1 h exposure to deltamethrin 0.05%

Deltamethrin 0.05%	*OR*	Lower	Upper	*α* = 0.05
GGTTCC/TTGGTT GGFFPP/VVCCSS	4.95	1.29	19.01	*
GGTTCC/TGTGTC GGFFPP/VGFCSP	6.37	1.91	21.18	*
TTGGTT/TGTGTC VVCCSS/VGFCSP	1.29	0.41	4.01	NS
TTGGTT/GGTTCC VVCCSS/GG/FF/PP	0.20	0.05	0.78	NS
TGTGTC/GGTTCC VGFCSP/GGFFPP	0.16	0.05	0.52	NS
TGTGTC/TTGGTT VGFCSP/VVCCSS	0.78	0.25	2.42	NS

*OR* = odds ratio with 95% confidence intervals; *significance, NS = not significant as confidence intervals encompass 1. Order of mutations is 1016/1534/989, upper genotype is the base configuration: T = wildtype, G or C = mutant; lower genotype is the amino acid configuration

Five genotypes (A, B, C/L, l, J) occurred in both survivors of DDT 4% and in the dead individuals (A, B, C/L, I, J) (Table 4). There was no significant difference (*α* = 0.05) in the odds of being alive after exposure to DDT 4% if carrying one genotype over any other (Additional file 1: Table S2).

### Codons 410 and 1763

Thirty-two sequences of 93–95 bp in length (30 from mosquitoes from Al Rawabi and two from Al Safa) were obtained for codon 410. Each sequence was wildtype at codon 410, coding for valine (V) (Additional file 1: Table S3). Ninety-three sequences of 116 bp in length were obtained for codon 1763 (47 from mosquitoes from Al Rawabi and 46 from Al Safa). Each sequence was identical and wildtype, coding for aspartic acid (D) at codon 1763 (Additional file 1: Table S4).

## Discussion

The consequences of insecticide resistance in insect vectors of disease have severe implications for human health. Multiple studies characterise mutations in the *Vssc* gene of the dengue vector, *Ae*. *aegypti*, around the world and attempt to relate mutation status to local resistance phenotype (for example, [3, 32, 37]) or, in other cases, to investigate physiological effects of the mutations, which leads to identification of those that play a causal role (for example, [12, 21]). Such studies are useful in aiding prediction about which types of pyrethroid insecticide might succeed or fail in controlling the mosquito if applied alongside knowledge of metabolic resistance status.

Our study takes a different approach in that after characterizing *Vssc* mutations in *Ae*. *aegypti* from two districts of Jeddah, we compare the mutations with those in *Ae*. *aegypti* from other world regions by analyzing as haplotypes and discuss their utility as both comparative markers in field and laboratory populations to ensure quality control for *Wolbachia* release programs and as indicators of whether resistance has developed by local selection or invasion by resistant individuals.

*Vssc* mutations at codons 989, 1016 and 1534 are present in *Ae*. *aegypti* from two districts of Jeddah representing potential future release sites for *Wolbachia* mosquitoes for control of dengue. The populations are not fixed for one genotype, but genotypes A, B, C are common, and no homozygote wildtype individuals were found in the sample. A wildtype haplotype exists, at least in Al Rawabi, so it is possible that homozygous wildtype individuals (genotype F) may occur in the population, albeit rarely. There is no indication that this haplotype still exists in Al Safa, but increased sampling might still find it. No instances of a mutation at codons 410 or 1763 were observed in the samples screened, indicating lack of independent selection or contact of the mosquito populations with those from Taiwan [24], Brazil [21], Mexico [23] or West and Central Africa [22].

The triple mutant homozygote (1016G/1534C/989P) (genotype K—Fig. 3) can now be confirmed from Al Safa and Al Rawabi districts, having been found previously only as a heterozygote in this region [5], suggesting that *Ae*. *aegypti* from Saudi Arabia may have a unique population history which could be further explored in a full genomic analysis. The implications of the triple mutant and genotypes I–L on pyrethroid resistance levels are not well understood, but their presence provides an opportunity to study effects of these genotypes in more detail in bioassays and fitness experiments. Although the triple mutant genotype is rare in the populations of *Ae*. *aegypti* sampled from Jeddah (2–3%), the frequency is higher than that reported from Myanmar (0.98%) [34] and is high enough to facilitate collection of adequate numbers to isolate individuals for crossing experiments which could allow further characterization of the H5 haplotype and I–L genotypes in bioassays. Two triple homozygous mutants were found in the live pool of our bioassay with permethrin 0.75%.

The *Vssc* genotypes found in Al Safa and Al Rawabi may all be constructed from haplotypes 1, 2, 3 and 5. Haplotype 4, found only in Taiwan of the countries sampled in the Indo-Pacific [28] and in Indonesia [3], is not found in the Saudi Arabian samples we have screened, which is consistent with results of Al Nazawi et al. [5]. It is possible that the triple heterozygous genotype in Saudi Arabia could be composed of either H1 + H2 (usual condition in the Indo-Pacific—genotype C) or H3 + H5 (triple wildtype plus triple mutant—genotype L) (Fig. 2). Both H3 and H5 are rare compared with H1 and H2, but there is a possibility that some of the heterozygotes have this rare configuration. It is not known whether there would be any difference in insecticide resistance in mosquitoes which have one or the other putative haplotype combination of the triple heterozygote.

Potential implications of the genotypes A–E and I–K on control of *Ae*. *aegypti* with pyrethroid insecticides have been surmised (Table 6) [5, 32, 38], but not all genotypes have been tested for effects on susceptibility. It is likely that efficacy of both Type I and Type II pyrethroids is compromised by many of these modifications to the *Vssc*, though genotypes have differential effects. As well as categorizing effects on resistance of each of these genotypes, a useful area of future research would be to pinpoint their fitness costs on *Ae*. *aegypti* both in wild and *Wolbachia*-infected populations.

**Table 6:** Pyrethroid resistance implications of voltage-sensitive sodium channel (*Vssc*) mutations in *Aedes aegypti* from the Kingdom of Saudi Arabia (Al Safa and Al Rawabi districts of Jeddah)

Genotype	V1016G	F1534C	S989P	Resistance implication	References
A	GG GG	TT FF	CC PP	Confers resistance to Type I and type II pyrethroids (HIGH LEVEL)	Plernsub et al. [32]
B	TT VV	GG CC	TT SS	Confers resistance to Type I pyrethroids (LOW LEVEL)	Plernsub et al. [32]
C	TG VG	TG FC	TC SP	Heterozygote (C)—confers low level of resistance to Type I and Type II pyrethroids (INTERMEDIATE LEVEL)	Plernsub et al. [32]
D	TG VG	TT FF	TC SP	Type II pyrethroids (practically SUSCEPTIBLE)	Plernsub et al. [32]
E	TT VV	TG FC	TT SS	May confer low level of resistance to Type I pyrethroids (not much higher than wildtype)	Plernsub et al. [32]
I	GG GG	TG FC	CC PP	Resistance level conferred by this genotype has not been fully ascertained—possibly susceptible to Type II pyrethroids	Al Nazawi et al. [5]
J	TG VG	GG CC	TC SP	Resistance level conferred by this genotype has not been fully ascertained—possibly susceptible to Type II pyrethroids	Al Nazawi et al. [5]
K	GG GG	GG CC	CC PP	Triple mutant homozygote—Extremely high resistance when created artificially in *Xenopus* oocytes—Resistance in nature not known. May have high fitness costs—possibly susceptible to Type II pyrethroids	Hirata et al. [38], Al Nazawi et al. [5]
L	TG VG	TG FC	TG SP	Putative triple heterozygote (L)—resistance level conferred, if any, is not known	–

Although resistance to three insecticides which target the *Vssc* was detected in bioassays in the *Ae*. *aegypti* from Jeddah in this study, the only clear association between *Vssc* mutations and resistance was established for the Type II pyrethroid, deltamethrin, with genotype A conferring a survival advantage compared with genotypes B (1534C) or C (triple heterozygote). Genotype A is known to reduce the sensitivity of the *Vssc* for both Type I and Type II pyrethroids [2], but in our study there was no obvious effect for Type I (permethrin 0.75%) or DDT. It may be that this lack of effect is concentration related and a different concentration of permethrin or DDT would reveal a segregation of genotypes between dead and surviving mosquitoes as reported from the Jazan region of Saudi Arabia [4]. In a similar study in *Ae*. *aegypti* from Malaysia [17], a survival advantage over wildtype was conferred to mosquitoes by genotypes B and C when exposed to permethrin 0.25%.

Comparison of *Vssc* mutations in *Ae*. *aegypti* throughout the world and their association with phenotypic resistance levels reveals that their effects have some dependence on context which may arise from differences in genetic background. It is important to note that target-site resistance may not be the only mechanism of resistance to pyrethroids that has been selected in the Jeddah populations of *Ae*. *aegypti*. A role of metabolic resistance in *Ae*. *aegypti* from Al Safa and Al Rawabi is likely. Al Nazawi et al. [5] saw an increase in mortality of *Ae*. *aegypti* from Jeddah with deltamethrin, using the synergist (PBO), which indicates that detoxification by oxidases (cytochrome P450 mixed function oxidase system) accounts for some of the pyrethroid resistance response. We also found an indication of such synergism in bioassays with the Type I pyrethroid, permethrin 0.75%.

To ensure similarity in genetic background in mosquitoes from the field in Jeddah with those containing *Wolbachia* and reared in the laboratory for release, a backcrossing scheme is being employed. Male mosquitoes from the field release sites are crossed with females from the *Wolbachia* colony. At least three rounds of backcrossing will be completed before the colony is closed for mass-rearing. The backcrossing has the function of introducing resistance mechanisms into the release population. As well as *Vssc* mutations and metabolic resistance mechanism against pyrethroids, mechanisms for resistance to other chemical groups would also be incorporated, if present. *Vssc* resistance allele status will be monitored in the release population, and further backcrossing will be carried out as required to maintain their frequency. Screening of *Vssc* mutations provides a point of reference for when another round of backcrossing would be beneficial, as we have shown that these mutations can be lost over time in a closed population [17].

## Conclusions

The continuing presence of the wildtype haplotype and minor difference in genotypes between mosquitoes in Al Safa and Al Rawabi suggest that selection for pyrethroid resistance may be patchy between districts in Jeddah. Changes in genotype frequencies over time also suggest that selection for pyrethroid resistance may be ongoing. A future area of our research, in which we look at fine-scale population genomics of *Ae*. *aegypti* in our study areas of Jeddah, will provide further information as to whether the resistance in the populations has arisen from local selection or by invasion of resistant mosquitoes from other regions. The presence of the triple mutant, which is only found in mosquito populations from a few regions throughout the world, suggests that local selection for resistance is likely in Jeddah. Our results provide preliminary insight into differences between *Ae*. *aegypti* populations in Jeddah and those from some other parts of the world. They add to the information available for ongoing monitoring of resistance particularly with implementation of resistance management programs and indicate *Vssc* genotypes required in *Wolbachia* mosquito release populations to ensure homogeneity with the target field population, a prerequisite for establishment and subsequent reductions in transmission of dengue.

## Supplementary Information

**Additional file 1: Table S1. **Frequency of *Vssc* mutations in dead and surviving *Ae*. *aegypti* mosquitoes from WHO insecticide paper bioassays with Type I pyrethroid, permethrin (0.75%) (OR = odds ratio with 95% confidence intervals) (order of mutations is 1016/1534/989, genotype given is the base configuration: T = wildtype, G or C = mutant). **Table S2.** Frequency of *Vssc* mutations in dead and surviving *Ae*. *aegypti* mosquitoes from WHO insecticide paper bioassays with DDT (4%) (OR = odds ratio with 95% confidence intervals) (order of mutations is 1016/1534/989, genotype given is the base configuration: T = wildtype, G or C = mutant). **Table S3.** DNA sequence around *Vssc* Domain I codon 410 in *Aedes* *aegypti* from Jeddah, Kingdom of Saudi Arabia. **Table S4.** DNA sequence around *Vssc* Domain IV codon 1763 in *Aedes* *aegypti* from Jeddah, Kingdom of Saudi Arabia (codon 1763 highlighted in yellow) aligned to GenBank sequences MK495874 (*Ae*. *aegypti* D1763—wildtype) and MK495875 (*Ae*. *aegypti* 1763Y—mutant).

## Data Availability

All data generated or analyzed during this study are included in this published article [and its additional information files].
