# Supplementary material for: Voltage-sensitive sodium channel (Vssc) mutations associated with pyrethroid insecticide resistance in Aedes aegypti (L.) from two districts of Jeddah, Kingdom of Saudi Arabia: baseline information for a Wolbachia release program
Source: Parasit Vectors. 2021 Jul 12;14:361. doi: 10.1186/s13071-021-04867-3 (PMC8273952; doi:10.1186/s13071-021-04867-3)
Supplement: Supplementary file 1 — Additional file 1: Table S1. Frequency of Vssc mutations in dead and surviving Ae. aegypti mosquitoes from WHO insecticide paper bioassays with Type I pyrethroid, permethrin (0.75%) (OR = odds ratio with 95% confidence intervals) (order of mutations is 1016/1534/989, genotype given is the base configuration: T = wildtype, G or C = mutant). Table S2. Frequency of Vssc mutations in dead and surviving Ae. aegypti mosquitoes from WHO insecticide paper bioassays with DDT (4%) (OR = odds ratio with 95% confidence intervals) (order of mutations is 1016/1534/989, genotype given is the base configuration: T = wildtype, G or C = mutant). Table S3. DNA sequence around Vssc Domain I codon 410 in Aedes aegypti from Jeddah, Kingdom of Saudi Arabia. Table S4. DNA sequence around Vssc Domain IV codon 1763 in Aedes aegypti from Jeddah, Kingdom of Saudi Arabia (codon 1763 highlighted in yellow) aligned to GenBank sequences MK495874 (Ae. aegypti D1763—wildtype) and MK495875 (Ae. aegypti 1763Y—mutant). [file 13071_2021_4867_MOESM1_ESM.docx]

**Additional File 1**

Table S1. Frequency of *Vssc* mutations in dead and surviving *Ae*. *aegypti* mosquitoes from WHO insecticide paper bioassays with Type I pyrethroid, permethrin (0.75%) (OR =Odds Ratio with 95% confidence intervals) (Order of mutations is 1016/1534/989, genotype given is the base configuration: T=wildtype, G or C = mutant)

|  | **GG/TT/CC** | **TT/GG/TT** | **TG/TG/TC** | **GG/TG/CC** | **TG/GG/TC** | **GG/GG/CC** |  |
| --- | --- | --- | --- | --- | --- | --- | --- |
| Alive | 12 | 5 | 20 | 0 | 1 | 2 | 40 |
| Dead | 11 | 12 | 16 | 0 | 1 | 0 | 40 |

| DDT 4% | **OR** | **LOWER** | **UPPER** | α=0.05 |
| --- | --- | --- | --- | --- |
| GGTTCC/TTGGTT | 2.62 | 0.70 | 9.86 | NS |
| GGTTCC/TGTGTC | 0.87 | 0.31 | 2.49 | NS |
| TTGGTT/TGTGTC | 0.33 | 0.10 | 1.14 | NS |
| TTGGTT/GGTTCC | 0.38 | 0.10 | 1.44 | NS |
| TGTGTC/GGTTCC | 1.15 | 0.40 | 3.27 | NS |
| TGTGTC/TTGGTT | 3.00 | 0.87 | 10.30 | NS |

Table S2. Frequency of *Vssc* mutations in dead and surviving *Ae*. *aegypti* mosquitoes from WHO insecticide paper bioassays with DDT (4%) (OR =Odds Ratio with 95% confidence intervals) (Order of mutations is 1016/1534/989, genotype given is the base configuration: T=wildtype, G or C = mutant)

|  | **GG/TT/CC** | **TT/GG/TT** | **TG/TG/TC** | **GG/TG/CC** | **TG/GG/TC** | **00/TT/00** | **TOTAL** |
| --- | --- | --- | --- | --- | --- | --- | --- |
| Alive | 10 | 13 | 13 | 3 | 1 | 0 | 40 |
| Dead | 7 | 11 | 16 | 1 | 4 | 1 | 40 |

| DDT 4% | **OR** | **LOWER** | **UPPER** | α=0.05 |
| --- | --- | --- | --- | --- |
| GGTTCC/TTGGTT | 1.21 | 0.34 | 4.24 | NS |
| GGTTCC/TGTGTC | 1.76 | 0.52 | 5.91 | NS |
| TTGGTT/TGTGTC | 1.45 | 0.49 | 4.31 | NS |
| TTGGTT/GGTTCC | 0.83 | 0.24 | 2.91 | NS |
| TGTGTC/GGTTCC | 0.57 | 0.17 | 1.91 | NS |
| TGTGTC/TTGGTT | 0.69 | 0.23 | 2.04 | NS |
| GGTTCC/GGTGCC | 0.48 | 0.04 | 5.58 | NS |
| GGTTCC/TGGGTC | 5.71 | 0.52 | 62.66 | NS |
| TTGGTT/GGTGCC | 0.39 | 0.04 | 4.35 | NS |
| TTGGTT/TGGGTC | 4.73 | 0.46 | 48.77 | NS |
| GGTGTC/GGTTCC | 2.10 | 0.18 | 24.60 | NS |
| GGTGTC/TTGGTT | 2.54 | 0.23 | 28.02 | NS |
| GGTGTC/TGTGTC | 3.69 | 0.34 | 39.84 | NS |
| GGTGTC/TGGGTC | 12.00 | 0.51 | 280.11 | NS |
| TGGGTC/GGTTCC | 0.18 | 0.02 | 1.92 | NS |
| TGGGTC/TTGGTT | 0.21 | 0.02 | 2.18 | NS |
| TGGGTC/TGTGTC | 0.31 | 0.03 | 3.10 | NS |
| TGGGTC/GGTGCC | 0.08 | 0.00 | 1.95 | NS |

Table S3. DNA sequence around *Vssc* Domain I codon 410 in *Aedes* *aegypti* from Jeddah, Kingdom of Saudi Arabia

32 sequences of 93-95 bp in length (30 from mosquitoes from Al Rawabi and two from Al Safa) were obtained for codon 410. Each sequence was wildtype at codon 410, coding for valine (V).

Consensus GTGGCACATGCTCTTCTTCATTGTGATTATCTTCTTGGGTTCGTTCTACCTTGTAAATTT 60

KY747529 extract V410 wt GUGGCACAUGCUCUUCUUCAUUGUGAUUAUCUUCUUGGGUUCGUUCUACCUUGUAAAUUU 60

KY747530.1 extract 410L mut GTGGCACATGCTCTTCTTCATTGTGATTATCTTCTTGGGTTCGTTCTACCTTTTAAATTT 60

RA043_aegSCR10.ab1 (reversed) GTGGCACATGCTCTTCTTCATTGTGATTATCTTCTTGGGTTCGTTCTACCTTGTAAATTT 60

RA069_aegSCR10.ab1 (reversed) GTGGCACATGCTCTTCTTCATTGTGATTATCTTCTTGGGTTCGTTCTACCTTGTAAATTT 60

RA107_aegSCR10.ab1 (reversed) GTGGCACATGCTCTTCTTCATTGTGATTATCTTCTTGGGTTCGTTCTACCTTGTAAATTT 60

RA110_aegSCR10.ab1 (reversed) GTGGCACATGCTCTTCTTCATTGTGATTATCTTCTTGGGTTCGTTCTACCTTGTAAATTT 60

RA114_aegSCR10.ab1 (reversed) GTGGCACATGCTCTTCTTCATTGTGATTATCTTCTTGGGTTCGTTCTACCTTGTAAATTT 60

RA120_aegSCR10.ab1 (reversed) GTGGCACATGCTCTTCTTCATTGTGATTATCTTCTTGGGTTCGTTCTACCTTGTAAATTT 60

RA146_aegSCR10.ab1 (reversed) GTGGCACATGCTCTTCTTCATTGTGATTATCTTCTTGGGTTCGTTCTACCTTGTAAATTT 60

RA153_aegSCR10.ab1 (reversed) GTGGCACATGCTCTTCTTCATTGTGATTATCTTCTTGGGTTCGTTCTACCTTGTAAATTT 60

RA157_aegSCR10.ab1 (reversed) GTGGCACATGCTCTTCTTCATTGTGATTATCTTCTTGGGTTCGTTCTACCTTGTAAATTT 60

RA169_aegSCR10.ab1 (reversed) GTGGCACATGCTCTTCTTCATTGTGATTATCTTCTTGGGTTCGTTCTACCTTGTAAATTT 60

RB052_aegSCR10.ab1 (reversed) GTGGCACATGCTCTTCTTCATTGTGATTATCTTCTTGGGTTCGTTCTACCTTGTAAATTT 60

RB071_aegSCR10.ab1 (reversed) GTGGCACATGCWCTTCTTCATTGTGATTATCTTCTTGGGTTCGTTCTACCTTGTAAATTT 60

RB105_aegSCR10.ab1 (reversed) GTGGCACATGCTCTTCTTCATTGTGATTATCTTCTTGGGTTCGTTCTACCTTGTAAATTT 60

RB111_aegSCR10.ab1 (reversed) GTGGCACATGCTCTTCTTCATTGTGATTATCTTCTTGGGTTCGTTCTACCTTGTAAATTT 60

RB124_aegSCR10.ab1 (reversed) GTGGCACATGCTCTTCTTCATTGTGATTATCTTCTTGGGTTCGTTCTACCTTGTAAATTT 60

RB128_aegSCR10.ab1 (reversed) GTGGCACATGCTCTTCTTCATTGTGATTATCTTCTTGGGTTCGTTCTACCTTGTAAATTT 60

RB152_aegSCR10.ab1 (reversed) GTGGCACATGCTCTTCTTCATTGTGATTATCTTCTTGGGTTCGTTCTACCTTGTAAATTT 60

RB157_aegSCR10.ab1 (reversed) GTGGCACATGCTCTTCTTCATTGTGATTATCTTCTTGGGTTCGTTCTACCTTGTAAATTT 60

RB163_aegSCR10.ab1 (reversed) GTGGCACATGCTCTTCTTCATTGTGATTATCTTCTTGGGTTCGTTCTACCTTGTAAATTT 60

RB171_aegSCR10.ab1 (reversed) GTGGCACATGCTCTTCTTCATTGTGATTATCTTCTTGGGTTCGTTCTACCTTGTAAATTT 60

RB172_aegSCR10.ab1 (reversed) GTGGCACATGCTCTTCTTCATTGTGATTATCTTCTTGGGTTCGTTCTACCTTGTAAATTT 60

RB175_aegSCR10.ab1 (reversed) GTGGCACATGCTCTTCTTCATTGTGATTATCTTCTTGGGTTCGTTCTACCTTGTAAATTT 60

RC045_aegSCR10.ab1 (reversed) GTGGCACATGCTCTTCTTCATTGTGATTATCTTCTTGGGTTCGTTCTACCTTGTAAATTT 60

RC065_aegSCR10.ab1 (reversed) GTGGCACATGCTCTTCTTCATTGTGATTATCTTCTTGGGTTCGTTCTACCTTGTAAATTT 60

RC103_aegSCR10.ab1 (reversed) GTGGCACATGCTCTTCTTCATTGTGATTATCTTCTTGGGTTCGTTCTACCTTGTAAATTT 60

RC104_aegSCR10.ab1 (reversed) GTGGCACATGCTCTTCTTCATTGTGATTATCTTCTTGGGTTCGTTCTACCTTGTAAATTT 60

RC142_aegSCR10.ab1 (reversed) GTGGCACATGCTCTTCTTCATTGTGATTATCTTCTTGGGTTCGTTCTACCTTGTAAATTT 60

RC145_aegSCR10.ab1 (reversed) GTGGCACATGCTCTTCTTCATTGTGATTATCTTCTTGGGTTCGTTCTACCTTGTAAATTT 60

RC147_aegSCR10.ab1 (reversed) GTGGCACATGCTCTTCTTCATTGTGATTATCTTCTTGGGTTCGTTCTACCTTGTAAATTT 60

RC158_aegSCR10.ab1 (reversed) GTGGCACATGCTCTTCTTCATTGTGATTATCTTCTTGGGTTCGTTCTACCTTGTAAATTT 60

SL149_aegSCR10.ab1 (reversed) GTGGCACATGCTCTTCTTCATTGTGATTATCTTCTTGGGTTCGTTCTACCTTGTAAATTT 60

SL160_aegSCR10.ab1 (reversed) GTGGCACATGCTCTTCTTCATTGTGATTATCTTCTTGGGTTCGTTCTACCTTGTAAATTT 60

Consensus GATCTTGGCCATTGTCGCCATGTCGTACGACGAAC 95

KY747529 extraction GAUCUUGGCCAUUGUCGCCAUGUCGUACGACGAAC 95

KY747530.1 extraction GATCTTGGCCATTGTCGCCATGTCGTACGACGAAC 95

RA043_aegSCR10.ab1 (reversed) GATCTTGGCCATTGTCGCCATGTCGTACGACGNN- 94

RA069_aegSCR10.ab1 (reversed) GATCTTGGCCA-TGTCGCCATGTCGTACGACGAA- 93

RA107_aegSCR10.ab1 (reversed) GATCTTGGCCATTGTCGCCATGTCGTACGACGAA- 94

RA110_aegSCR10.ab1 (reversed) GATCTTGGCCA-TGTCGCCATGTCGTACGACGAA- 93

RA114_aegSCR10.ab1 (reversed) GATCTTGGCCATTGTCGCCATGTCGTACGACGAA- 94

RA120_aegSCR10.ab1 (reversed) GATCTTGGCCATTGTCGCCATGTCGTACGACGAA- 94

RA146_aegSCR10.ab1 (reversed) GATCTTGGCCATTGTCGCCATGTCGTACGACGAA- 94

RA153_aegSCR10.ab1 (reversed) GATCTTGGCCATTGTCGCCATGTCGTACGACGAA- 94

RA157_aegSCR10.ab1 (reversed) GATCTTGGCCATTGTCGCCATGTCGTACGACGAA- 94

RA169_aegSCR10.ab1 (reversed) GATCTTGGCCATTGTCGCCATGTCGTACGACGAA- 94

RB052_aegSCR10.ab1 (reversed) GATCTTGGCCA-TGTCGCCATGTCGTACGACGAA- 93

RB071_aegSCR10.ab1 (reversed) GATCTTGGCCA-TGTCGCCATGTCGTACGACGAA- 93

RB105_aegSCR10.ab1 (reversed) GATCTTGGCCATTGTCGCCATGTCGTACGACGAA- 94

RB111_aegSCR10.ab1 (reversed) GATCTTGGCCATTGTCGCCATGTCGTACGACGAA- 94

RB124_aegSCR10.ab1 (reversed) GATCTTGGCCATTGTCGCCATGTCGTACGACGAA- 94

RB128_aegSCR10.ab1 (reversed) GATCTTGGCCATTGTCGCCATGTCGTACGACGAA- 94

RB152_aegSCR10.ab1 (reversed) GATCTTGGCCA-TGTCGCCATGTCGTACGACGAA- 93

RB157_aegSCR10.ab1 (reversed) GATCTTGGCCATTGTCGCCATGTCGTACGACGAA- 94

RB163_aegSCR10.ab1 (reversed) GATCTTGGCCATTGTCGCCATGTCGTACGACGAA- 94

RB171_aegSCR10.ab1 (reversed) GATCTTGGCCA-TGTCGCCATGTCGTACGACGAA- 93

RB172_aegSCR10.ab1 (reversed) GATCTTGGCCA-TGTCGCCATGTCGTACGACGAA- 93

RB175_aegSCR10.ab1 (reversed) GATCTTGGCCATTGTCGCCATGTCGTACGACNA-- 93

RC045_aegSCR10.ab1 (reversed) GATCTTGGCCATTGTCGCCATGTCGTACGACGAA- 94

RC065_aegSCR10.ab1 (reversed) GATCTTGGCCATTGTCGCCATGTCGTACGACGAA- 94

RC103_aegSCR10.ab1 (reversed) GATCTTGGCCA-TGTCGCCATGTCGTACGACGAA- 93

RC104_aegSCR10.ab1 (reversed) GATCTTGGCCATTGTCGCCATGTCGTACGACGAA- 94

RC142_aegSCR10.ab1 (reversed) GATCTTGGCCATTGTCGCCATGTCGTACGACGAA- 94

RC145_aegSCR10.ab1 (reversed) GATCTTGGCCATTGTCGCCATGTCGTACGACGAA- 94

RC147_aegSCR10.ab1 (reversed) GATCTTGGCCA-TGTCGCCATGTCGTACGACGAA- 93

RC158_aegSCR10.ab1 (reversed) GATCTTGGCCA-TGTCGCCATGTCGTACGACGAA- 93

SL149_aegSCR10.ab1 (reversed) GATCTTGGCCA-TGTCGCCATGTCGTACGACGAA- 93

SL160_aegSCR10.ab1 (reversed) GATCTTGGCCA-TGTCGCCATGTCGTACGACGAA- 93

Table S4. DNA sequence around *Vssc* Domain IV codon 1763 in *Aedes* *aegypti* from Jeddah, Kingdom of Saudi Arabia (codon 1763 highlighted in yellow) aligned to GenBank sequences MK495874 (*Ae*. *aegypti* D1763 - wildtype) and MK495875 (*Ae*. *aegypti* 1763Y – mutant)

93 sequences of 116 bp in length were obtained for codon 1763 (47 from mosquitoes from Al Rawabi and 46 from Al Safa). Each sequence was identical and wildtype. Only one representative sequence is shown.

Consensus ACATCTGTCTGCTGCTGTTCTTGGTCATGTTCATCTTCGCCATCTTCGGCATGTCGTTCT 60

20201118KSA4_F10_albSCF7.ab1 ---TCTGTCTGCTGCTGTTCTTGGTCATGTTCATCTTCGCCATCTTCGGCATGTCGTTCT 57

MK495874 D1763 ACATCTGTCTGCTGCTGTTCTTGGTCATGTTCATCTTCGCCATCTTCGGCATGTCGTTCT 60

MK495875 1763Y ACATCTGTCTGCTGCTGTTCTTGGTCATGTTCATCTTCGCCATCTTCGGCATGTCGTTCT 60

Consensus TCATGCACGTGAAGGACAAGAGCGGGCTGGACGATGTGTACAATTTCAAGACGTTCGGCC 120

20201118KSA4_F10_albSCF7.ab1 TCATGCACGTGAAGGACAAGAGCGGGCTGGACGATGTGTACAATTTCAAGACGTTCGGC- 117

MK495874 D1763 TCATGCACGTGAAGGACAAGAGCGGGCTGGACGATGTGTACAATTTCAAGACGTTCGGCC 120

MK495875 1763Y TCATGCACGTGAAGTACAAGAGCGGGCTGGACGATGTGTACAATTTCAAGACGTTCGGCC 120
